# Murdered by Allopathy

**Published:** 1894-08

**Authors:** 


### "MURDERED BY ALLOPATHY."

The following letter was written by a gentleman who is engaged in the cultivation of oranges in Florida. He is not a graduate in medicine as would be supposed from his style, but an enthusiast upon Homœopathy and accustomed to administering it among his neighbors.

It is so instructive to the student of Homœopathy that it is considered a suitable addition to the pages of this journal.

FLORIDA, July 14th, 1894.

DEAR MRS. B.—My wife has been writing a description of the ordeal through which we have just passed. It does not give a correct view of the case, and thinking that you would like to know more about it I give my version. My son came home from his work in the mines with a severe attack of remittent fever and a painful diarrhœa. After I had been treating him with homœopathic remedies for nine days the fever changed to an intermittent, became less severe and terminated in a most profuse sweat. On the ninth day the diarrhœa had become painless. He was, as I fondly believed, on the road to returning health. He, however, was not satisfied because the diarrhœa continued, and insisted on sending for an allopathic doctor. The doctor came and said he was getting well nicely, but that the diarrhœa should be stopped. So he gave an astringent and next day the looseness was stopped. The day after that Edwin began to show such symptoms as sickness at the stomach, anxiety, and declarations that he never would get well. Pains in the stomach and bowels then set in, followed by an attack of dysentery, with bloody mucus, and flakes of epithelium. I at once set about treating the patient with *Mercurius-corr.* and Colocynthis. In about six hours the tenesmus had gone, the excrementitious matter had returned, and the discharges became painless. But an inflammation of the stomach now set in which seemed to be relieved with Nux Vomica. The next day after that symptoms of mania set in, which increased till he became violent and it took several men to hold him in bed. We had two doctors who both advised us to send him to the asylum, as they were satisfied that he would develop into a maniac. Steps were taken to this end, but by the time he could be sent he became rational and all the symptoms had disappeared. So the doctors continued to treat him for some days, and he appeared to be getting well. The looseness continued, however, but to my mind it was salutary and necessary, and it was withal painless and not more frequent than say about six times in twelve hours. Now the doctors insisted on giving an astringent. I protested with all my might against it. The doctors commanded me not to interfere either to give any of my medicines or omit those they gave. I was so weak and faint from anxiety, loss of sleep, etc., etc., and moreover the only man who could be depended upon as a nurse joined with the doctors, and declared that if I interfered he would go home. I protested that his chances would be better if he were set up as a mark to be shot at at ten rods than if he continued this treatment. Next day his brain became very active. About eight hours after that the fatal dose was given which caused him to relapse into aphasia. Then came acute mania. He was speechless and so violent that we had to strap him down to the cot upon which he lay. He struggled violently till he died—*murdered by Allopathy*. I am in such a state of weakness and prostration that writing this letter gives me a violent headache and I must close.

Affectionately yours,

